# Characteristic Radiological findings in Preterm Infants with Missed Intestinal Perforation

**Published:** 2014-07-10

**Authors:** Sivasankar Jayakumar, Nitin Patwardhan

**Affiliations:** Department of Paediatric surgery, University Hospitals of Leicester Infirmary road, Leicester, United Kingdom LE1 5WW

**Keywords:** Missed intestinal perforation, Pseudocyst, Necrotizing enterocolitis, Preterm, Neonate

## Abstract

BACKGROUND: Pneumoperitoneum on radiological imaging is typical in intestinal perforation in necrotizing enterocolitis [NEC]. However, it is not seen in all cases and intestinal perforation is missed on occasions. We present a series of preterm infants with characteristic x-ray findings that on exploration revealed missed intestinal perforation.

METHODS: Retrospective review of neonates with intra-operative diagnosis of intestinal perforation which was missed on x-ray abdomen over a period of 6 months is being presented here.

RESULTS: Three neonates born at 24 (24-30) weeks of gestation were identified. PDA was noted in all 3 patients and they required ventilator and inotropic support. Feeds were commenced at 5 (2-7) days of life. All three patients were treated for NEC. Surgical opinion was sought in view of localized gas shadow in a fixed position seen on repeated x-rays in all three patients. All three patients had laparotomy and small bowel resection with ileostomy formation at a mean age of 26 (24-46) days. Intra-operatively, small bowel perforation and adjacent pseudocysts filled with air and intestinal contents were noted in all 3 patients. Post-operatively full feeds were established in all patients.

CONCLUSION: In premature infants with NEC, intestinal perforation can be missed on occasions. Our patients interestingly, developed characteristic abdominal x-ray findings that in our experience should prompt for surgical intervention.

## INTRODUCTION

Necrotizing enterocolitis (NEC) is a severe inflammatory process of the gastrointestinal tract in neonates and is associated with a significant morbidity and mortality. Despite advances in neonatal care, NEC still remains as one of the most common causes of surgical intervention in the neonate. In NEC, the extent of disease could be focal [Isolated], multifocal or pan-intestinal. In a retrospective study on 32 patients with NEC, isolated NEC was present in 8 (25%), multifocal NEC in 19 (59%) and pan intestinal NEC in 5 (16%) of the operated cases [1]. Abdominal x-ray [AXR] is an important component of the assessment of infants with suspected NEC. AXR findings in NEC ranges from dilated bowel loops in suspected NEC to pneumatosis intestinalis, portal venous gas shadows and pneumoperitoneum in cases of definite NEC [2]. Pneumoperitoneum is widely accepted as an indication for surgical intervention. Very rarely, such a pneumoperitoneum secondary to intestinal perforation could be missed. We present a series of three preterm neonates who had initial treatment for NEC and later developed characteristic AXR findings that on surgical intervention were noted to be pseudocysts adjacent to bowel perforation. The details of presentation of these cases along with intra-operative findings and outcomes are presented and discussed in this article.

## MATERIALS AND METHODS

Retrospective review of neonates with intra-operative diagnosis of intestinal perforation which was missed on x-ray abdomen over a period of 6 months is being presented here.

## RESULTS

Three neonates, with a median gestational age of 24 (24-30) weeks with pseudocyst formation [noted at laparotomy] were identified over a period of 6 months. Antenatal scans did not reveal any bowel abnormality and antenatal serology was negative for HIV, Hepatitis B, VDRL and Rubella was immune in all 3 patients. PDA was noted in all 3 patients and they required inotropic support. Feeds were commenced at 5 [2, 5 and 7] days of life. Intravenous antibiotics for suspected NEC were commenced at 18 [14, 18 and 37] days of life. Surgical opinion was sought in view of persistent abdominal distention and ongoing sepsis. AXR at the time of referral did not show any definite signs of NEC in all 3 cases. Case 1, demonstrated a gas filled blister on the anterior abdominal wall [Fig. 1] at the time of referral. AXR in Case 2 showed persistent fixed loop surrounding a cyst like space in the abdomen [Fig. 2]. In Case 3, persistent localized gas shadow was seen on subsequent x-rays [Fig. 3].

**Figure:**
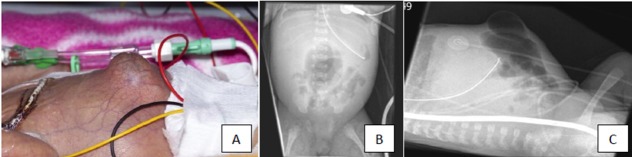
Fig. 1(A): Photograph of case 1 on day 23 of life, showing right peri-umbilical swelling;(B): AXR of Case 1 on day 23 of life showing localized gas shadow at the site of peri-umbilical swelling; (C): Lateral x-ray of the abdomen of case 1 on day 23 of life showing dilated bowel and air filled cyst at the site of peri-umbilical swelling.

**Figure:**
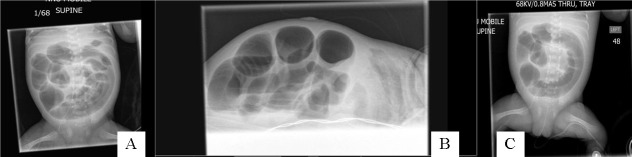
Figure 2: (A): AXR of Case 2 on day 44 of life, showing dilated bowel loop surrounding a cyst like appearance in the central abdomen; (B): Lateral shoot through x-ray of the abdomen of case 2 on day 44 of life showing dilated bowel in the central abdomen. No free air is seen; (C): AXR of Case 2 on day 46 of life, showing fixed dilated bowel loop surrounding a cyst like appearance in the central abdomen.

**Figure:**
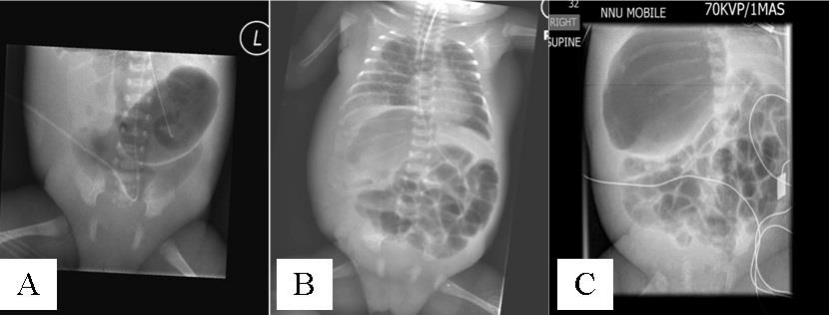
Figure 3: (A): AXR of case 3 done on day 14 of life, taken at the referring hospital showing free air in the abdomen. This finding was missed at the referring hospital; (B): AXR of case 3 done on day 24 of life, showing localized gas shadow in the right upper quadrant; (C): AXR of case 3 done on 26 of life, showing persistent localized gas shadow in the right upper quadrant.

All the three neonates underwent laparotomy and small bowel resection with ileostomy formation. The age of the patients at the time of laparotomy was 26 (24, 26 and 46) days. The individual patient characteristics of all 3 patients are summarized in Table 1. Intra-operatively, small bowel perforation along with pseudocyst containing air and intestinal contents was noted. No intestinal atresia or other bowel pathology was noted. Histopathological examination of the resected cysts and adjacent small bowel were consistent with NEC in all the three cases. Subsequent cystic fibrosis screening was negative in all three patients. Post-operatively full feeds were established in all 3 patients after stoma reversal and have been discharged home.

**Figure:**
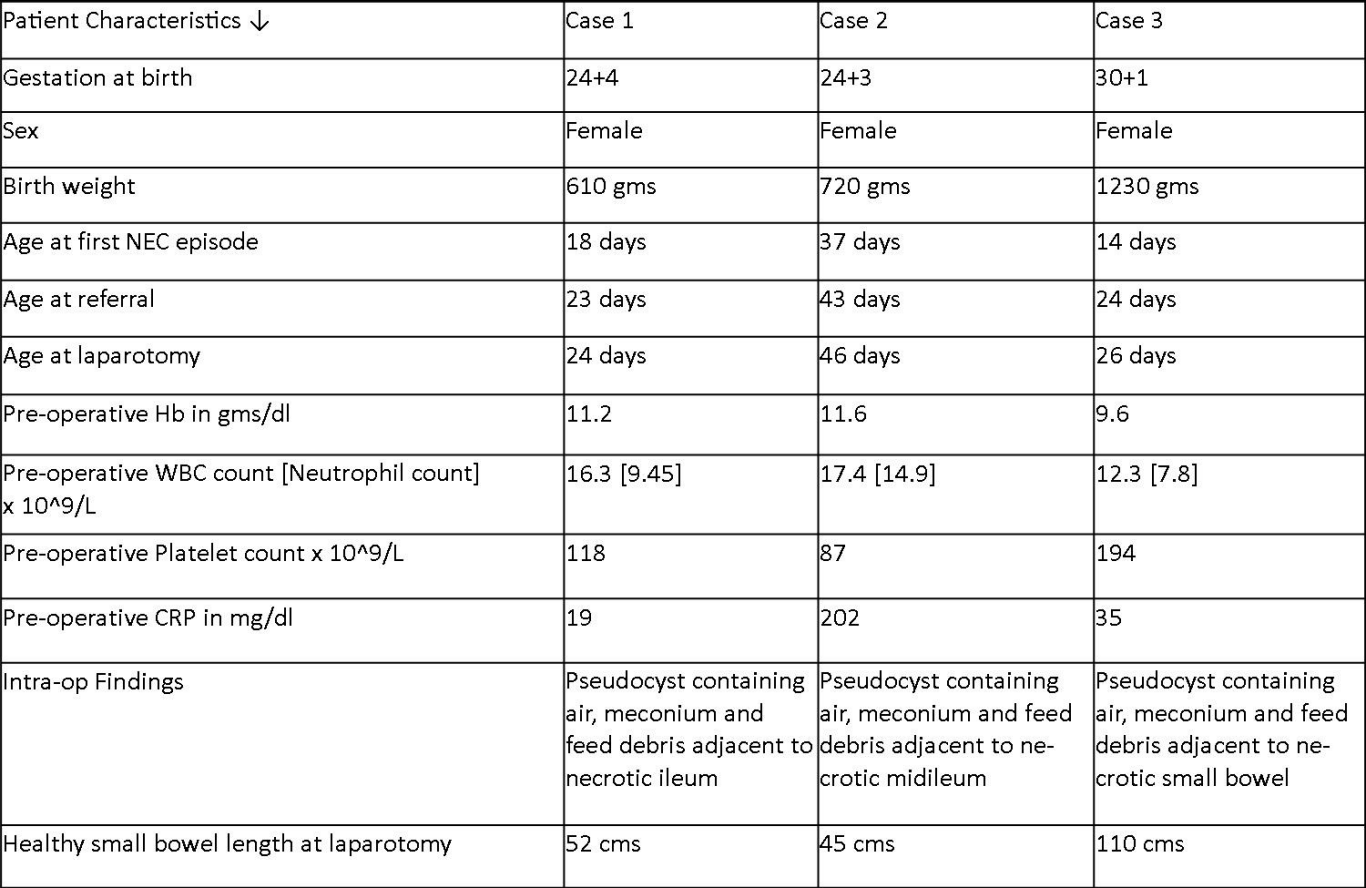
Table 1: Tabular column showing individual patient characteristics in all the three cases noted in our series.

## DISCUSSION

Necrotizing enterocolitis (NEC) is diagnosed in 6% of very low birth weight (VLBW; less than 1500 g) and 8% of extremely low birth weight (ELBW; less than 1000 g) infants [3]. Very few clinical studies have examined surgical findings in ELBW neonates with NEC. Earlier series on 50 ELBW patients showed focal NEC in 52% and pan involvement in 28%, but no cysts were reported in their study [1]. In a recent study on 158 ELBW neonates, the incidence of perforated NEC in ELBW premature infants was reported as 5.1% and the average age at onset of perforated NEC was 26 days [4]. In this study, dilated and fixed bowel loops, bowel wall thickening and ascites with brown stool-like substance on percutaneous drainage were the predominant signs at the time of diagnosis of perforated NEC and these signs may be considered as a significant sign of perforated NEC despite the absence of free air on radiography [4]. However, in the absence of free air on radiography and prior to percutaneous drainage, it is extremely difficult to know if bowel perforation has occurred. 

Although absolute evidence of bowel perforation/pneumoperitoneum was not evident in all our patients at the time of presentation to us, one of our patients [Case 3] had an AXR sent to us from the referring hospital that clearly revealed a pneumoperitoneum on day 14 of life [Fig. 3]. This patient was referred to us on day 24 of life after the above finding was missed at the referring hospital. The subsequent AXR taken on day 24 of life showed a cystic appearance but no obvious pneumoperitoneum. As similar findings were noted intra-operatively in the other 2 cases in our series with the background of NEC, it is likely that they also had a missed intestinal perforation and subsequent pseudocyst formation. 

Lorimer and Ellis classified meconium peritonitis resulting from antenatal perforation into generalized, fibro-adhesive, cystic, and healed types. In the cystic type, the perforation site does not heal effectively, and a thick-walled cyst forms by fixation of bowel loops [5]. Such cysts are usually referred as meconium pseudocysts. Although, in our series of three patients similar types of cysts were noted, none of these patients had an antenatal diagnosis of meconium cysts or any bowel abnormality suggesting a postnatal and not antenatal event in formation of the cysts. All patients had received a full course of triple antibiotics with presumed diagnosis of NEC prior to surgical referral. It is likely that the antibiotic support prevented generalized sepsis and may have contributed to localized pseudocyst formation that is similarly seen in antenatal meconium pseudocysts.

Wexler, in 1978, first described five cases of NEC with “persistent dilated loop sign” noted in plain abdominal x-rays. The position of dilated bowel loops in these cases remained unchanged in the abdominal x-rays over a period of 24 to 36 hours and he suggested that fixed loop of bowels should be surgically intervened [6]. However, a review of the records and radiographs of 21 newborn infants with NEC and 10 control infants performed by Leonard et al, to test the significance of the persistent loop sign in NEC did not support the use of a persistent segmental loop of bowel as a single criterion for surgical intervention [7]. Interestingly, all of our 3 patients had persistent fixed bowel loops with abnormal gas shadows on AXR which intra-operatively were confirmed to be extra intestinal.

It is well known that neonatal intestinal perforation may heal spontaneously with or without a drain insertion [8], however, in all other cases intervention is needed due to continued clinical deterioration of the patient. Our series highlights a third possible outcome for neonatal intestinal perforation. The cysts noted in our patients intra-operatively seem to be a rare occurrence and although we are unable to describe the exact pathogenesis behind them, they are most likely to represent a concealed intestinal perforation. However, such characteristic x-ray findings in premature neonates treated for NEC should prompt a caring physician/surgeon for surgical intervention.

## Conclusion

To conclude, intestinal perforation in premature infants with NEC is a well-known occurrence. This may be missed occasionally. The patient may continue to deteriorate or may have the perforation sealed. Our patients interestingly, developed pseudocysts localized at the site of perforation with characteristic abdominal x-ray findings that in our experience should prompt for surgical intervention.

## Footnotes

**Source of Support:** Nil

**Conflict of Interest:** None

